# Non-coding RNAs in oral cancer: Emerging biomarkers and therapeutic frontier

**DOI:** 10.1016/j.heliyon.2024.e40096

**Published:** 2024-11-02

**Authors:** Mehrdad Hashemi, Saloomeh Khoushab, Mina Hobabi Aghmiuni, Saeid Nemati Anaraki, Mina Alimohammadi, Afshin Taheriazam, Najma Farahani, Maliheh Entezari

**Affiliations:** a Department of Genetics, Faculty of Advanced Science and Technology, Tehran Medical Sciences, Islamic Azad University, Tehran, Iran; b Farhikhtegan Medical Convergence Sciences Research Center, Farhikhtegan Hospital Tehran Medical Sciences, Islamic Azad University, Tehran, Iran; c Department of Operative, Faculty of Dentistry, Tehran Medical Sciences, Islamic Azad University, Tehran, Iran; d Department of Immunology, School of Medicine, Shahid Beheshti University of Medical Sciences, Tehran, Iran; e Department of Orthopedics, Faculty of Medicine, Tehran Medical Sciences, Islamic Azad University,Tehran, Iran

**Keywords:** Transcriptome, OSCC, circRNA, miRNA, lncRNA, snoRNA, piRNA, Metabolomics reprogramming, Prognosis, ncRNAs, biomarkers

## Abstract

Around the world, oral cancer (OC) is a major public health problem, resulting in a significant number of deaths each year. Early detection and treatment are crucial for improving patient outcomes. Recent progress in DNA sequencing and transcriptome profiling has revealed extensive non-coding RNAs (ncRNAs) transcription, underscoring their regulatory importance. NcRNAs influence genomic transcription and translation and molecular signaling pathways, making them valuable for various clinical applications. Combining spatial transcriptomics (ST) and spatial metabolomics (SM) with single-cell RNA sequencing provides deeper insights into tumor microenvironments, enhancing diagnostic and therapeutic precision for OC. Additionally, the exploration of salivary biomarkers offers a non-invasive diagnostic avenue. This article explores the potential of ncRNAs as diagnostic and therapeutic tools for OC.

## Graphical abstract

**Figure:**
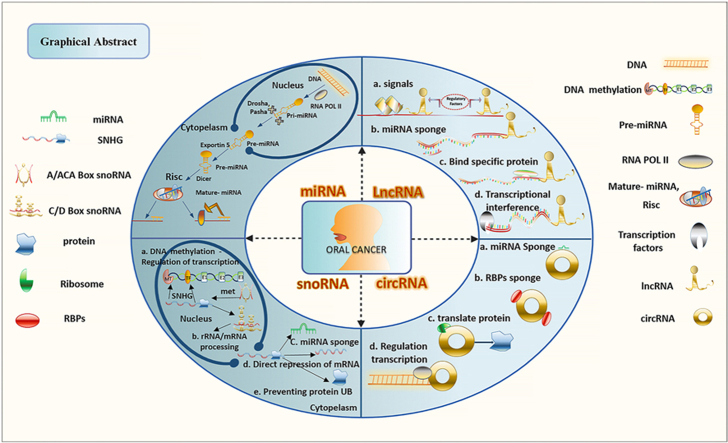

## 1 Introduction

**Figure 1:**
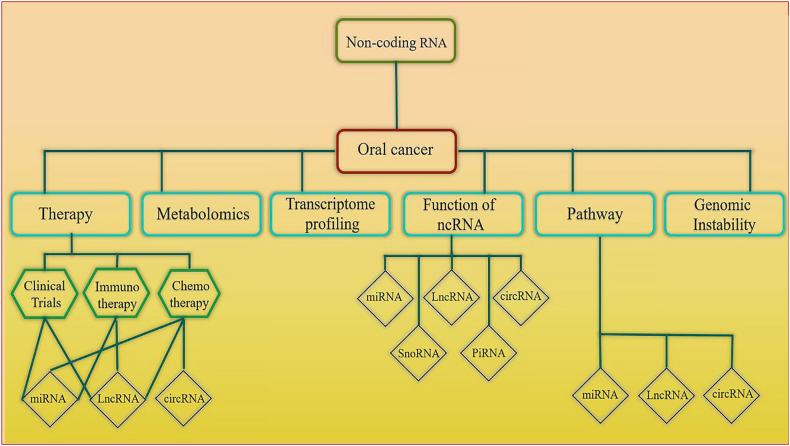
This flowchart summarizes ncRNAs in OC based on the current article.

Cancer, a serious public health concern, is recognized as the most common cause of mortality worldwide, responsible for about ten million deaths in 2020 [1]. Oral squamous cell carcinoma (OSCC), which is the most frequency form of oral cancer (OC), is the sixteenth most common cancer in the world, with over 200,000 new diagnoses each year. OSCC is classified into three subsites: buccal mucosal SCC (BMSCC), tongue SCC (TSCC), and lip SCC (LSCC) [[2], [3], [4]]. In the United States, in 2024, new cancer cases and deaths by gender for the oral cavity and pharynx are estimated. The estimated deaths are 8700 for males and 3530 for females [5]. The death rates of OC remain high, mainly due to delayed access to care, particularly in rural areas, reducing overall survival rates to around 50% [6]. OC is most common in the buccal mucosa, with the highest proportion occurring in the gingiva, floor, vestibule, palate, lower lip, tongue, and lower lip [7,8]. Several factors increase the risk of OC, including age, genetics, and lifestyle habits. Age, genetics, and gender all play a role, along with exposure to tobacco, alcohol, and certain viruses like HPV and EBV [9,10]. Although the prevalence of OC is high, there are no biomarkers for diagnosis, and disease diagnosis often occurs in the adnanced stages. Due to the delay in early-detection, the 5-year overall survival rate of patients is reflected in approximately 50%. Therefore, to find new biomarkers that have diagnostic, prognostic, and therapeutic potential in OC, a deeper understanding of the molecular mechanisms is required. Non-coding RNA (ncRNA) is one of the most promising classes of biomarkers for cancer diagnosis due to its involvement in almost all cellular functions. Around 70% of the human genome is transcribed into mRNA, with only about 2% being protein-coding, indicating that most ncRNA serve regulatory functions [[11], [12], [13]]. NcRNAs, which include transfer RNAs (tRNAs), ribosomal RNAs (rRNAs), microRNAs (miRNAs), small interfering RNAs (siRNAs), small nuclear RNAs (snRNAs), long non-coding RNAs (lncRNAs), and circular RNAs (circRNAs), are all valuable diagnostic tools and therapeutic targets [[14], [15], [16]]. Advances in DNA sequencing revealed that 80% of our genome can be transcribed into ncRNAs, which are vital for gene regulation and cellular processes and abnormal expression linked to diseases [[17], [18], [19]]. Given the limitations of current treatments for OSCC, which primarily rely on surgery with additional radiation and chemotherapy, there is a need for more research on early-stage OSCC detection [20,21]. Researchers are exploring ncRNAs as potential tools for diagnosing and treating OC due to their stability, specific tumor expression, and PCR detection [22,23]. Saliva provides a valuable resource for studying various biomolecules, including tumor cells, exosomes, antigens, and proteins, expanding our understanding of OC and related conditions [[24], [25], [26]]. This review explores OC, its epidemiology, risk factors, genomic instability, treatment options, and the potential of ncRNAs as diagnostic and therapeutic tools. In addition, the current study highlights the key roles of all ncRNAs in OC (figure. 1).

## 2 Genomic instability and OC

Microsatellite instability (MSI) and other minor structure variations like higher rates of base pair mutations, are examples of genomic instability. On the other hand, chromosomal instability (CIN), also known as significant structure variation, refers to alterations in chromosome number or structure [[27], [28], [29]]. Changes in the number and shape of chromosomes are caused by chromosomal instability (CIN), which is the most common type of genomic instability [30].

The majority of cancer cells have genomic instability. There is a greater propensity for genomic modification during cell division. Damage to several genes that regulate cell division and tumor suppressors frequently leads to cancer. Numerous surveillance mechanisms, including DNA damage checkpoint [[31], [32], [33]], DNA repair pathways (including mismatch repair (MMR), base excision repair (BER), nucleotide excision repair (NER), DNA double-strand break repair (DSBR)) [[34], [35], [36], [37], [38]], mitotic checkpoint [[39], [40], [41]], and telomere maintenance [[42], [43], [44]] are known to closely monitor genomic integrity [45].

As anticipated by the mutation hypothesis, genomic instability causes cancer development in hereditary malignancies by resulting from mutations in DNA repair genes. The molecular basis of genomic instability in sporadic (non-hereditary) cancers remains unknown [46].

Any technique that can identify chromosomal, microsatellite, or nucleotide alterations can quantify a component of genomic instability because genomic instability happens at numerous genetic levels [47]. Table 1 lists some of these methods.

**Table 1:** A number of methods to identify genomic instabilities

Approaches	Method	Ability to identify	reference
Multicellular	Array-CGH	Whole and segmental CIN	[48]
	SNP arrays	Whole and segmental CIN, SNP, UPD, LOH	[49]
	Flow cytometry	Cell ploidy/aneuploidy	[50]
	Whole-genome sequencing	Whole and segmental CIN, translocations, insertions, deletions, and mutations	[51]
	PCR	MSI, mitochondrial instability	[52,53]
Single-cell	Karyotyping	Whole and segmental CIN, aneuploidy	[54]
	FISH	assessment of CIN	[55]
	Single-cell sequencing	Whole and segmental CIN, translocations, insertions, deletions, and mutations	[56]

The karyotypes of OSCC comprise many numerical and structural anomalies, such as deletions, balanced and unbalanced translocations, isochromosomes, dicentric chromosomes, and homogeneously stained areas. These karyotypes are complicated, frequently approaching triploid [57]. Bhattacharya et al. employed comparative genomic hybridization (CGH) to identify a broad spectrum of genomic copy number abnormalities in oral dysplasia and SCC groups to shed light on the function of genomic aberrations in developing and spreading OC. Additionally, they discovered a highly significant correlation between the problematic neck lymph node status and 3q8pq20 status [58].

## 3 Metabolomics reprogramming and transcriptome profiling in OC

Metabolomics techniques like nuclear magnetic resonance (NMR) spectroscopy and mass spectrometry (MS) are being used to identify biomarkers for diagnosing OSCC, using various biological samples such as plasma, serum, saliva, urine, tissue, derivative cell lines, and animal models and examining RNA expression in OSCC-derived cell lines [59,60]. Cancer cells reprogram their metabolism to fuel their rapid growth and survival. Amino acid metabolism is a crucial aspect of this reprogramming, with current studies concentrating on the mechanisms through which lncRNAs govern this phenomenon. Unlike the well-known Warburg effect (focusing on glucose), lncRNAs appear to influence amino acid metabolism by affecting the expression of key enzymes and transporters. LncRNAs can regulate gene expression at multiple levels, making them powerful tools for cancer cells [61]. Miao Y et al., in a 2024 study on OC stem cells (CSCs) of single-cell RNA sequencing data from six OC patients, revealed their unique metabolic properties through a combined analysis of gene activity and metabolite levels, single-cell sequencing, and revealing that oral CSCs are less metabolically active than differentiated cancer cells, potentially making them resistant to current therapies targeting fast-growing tumors [62]. Analyzing gene activity across an entire organism (transcriptome-wide gene expression profiling) has been instrumental in revealing the molecular underpinnings of how diseases progress (prognosis) and drug sensitivity [63,64]. Transcriptome profiling can detect genetic events, including mutations, gene fusions, structural variants, and single nucleotide variants (SNVs). RNA-seq's high coverage allows for identifying germline polymorphisms and somatic mutations in genes with moderate to high expression levels, though combining genomic and transcriptomic sequencing yields the highest sensitivity and specificity [[65], [66], [67], [68], [69], [70], [71], [72], [73], [74]]. Transcriptome profiling is beneficial for finding gene fusions, which different types of genomic rearrangements can cause. While transcriptome profiling is less reliable for detecting mutations than DNA sequencing, it focuses on mutations in coding regions, which are more likely to be relevant to cancer development. Additionally, transcriptome profiling allows researchers to study allele-specific expression, which can reveal how genes are regulated and how mutations might affect cancer progression [75,76]. Cancer cells harbor both DNA mutations and changes in gene activity. Analyzing both the genome (DNA) and transcriptome (RNA) of a tumor provides a more comprehensive picture of the disease, revealing mutations and how those mutations affect which genes are turned on or off [77,78]. Zhang et al. used a bioinformatic pipeline to analyze RNA from two tumor samples and healthy ones, revealing 70,472 mutations in protein-coding regions. They identified 515 genes with significant mutations and 156 genes affecting function. Mutated genes were enriched in cell adhesion pathways and showed altered activity, highlighting RNA sequencing's usefulness in OSCC detection [79]. Researchers can gain insight into OSCC by examining metabolites and gene expression patterns, allowing them to uncover new diagnostic tools or treatment targets.

### 3.1 Function of MiRNA in OC

Cancer cells typically have too few tumor suppressor miRNAs and too many oncogenic miRNAs (oncomiRs). Depending on the cancer and specific miRNA, changes in miRNA levels can significantly impact cancer cell invasion, progression, and survival. In some cases, tumors rely on a specific oncomiR; blocking it can make the tumor disappear entirely [80,81]. However, miRNA effects can be complex. Some miRNAs can act as tumor suppressors in one situation, but as cancer promoters in another due to the many genes, a single miRNA can influence. Genetic alterations, such as chromosomal abnormalities and epigenetic modifications, can impact miRNA production in cancer [82,83].

Additionally, epigenetic silencing and abnormal transcription factor activity can contribute to the dysregulation of particular miRNAs in cancer. For instance, the tumor suppressor p53 can regulate the expression of several miRNAs, including miR-34, miR-29, and miR-15a/miR-16-1, implicating its role in OC development through miRNA modulation [84,85]. Oncogenic miRNAs are upregulated (expressed more) in cancer cells, while suppressive miRNAs are down-regulated (expressed less).

Interestingly, miR-21, frequently upregulated oncogenic miRNA, was identified across various cancers, including breast cancer, OSCC, and gastric cancer [[86], [87], [88], [89]]. Researchers have found that specific miRNAs play a role in developing and spreading OSCC. These miRNAs can suppress a tumor suppressor gene called PTEN (phosphatase and tensin homolog deleted on chromosome 10), which generally regulates cell death, migration, and invasion [[90], [91], [92]]. By inhibiting PTEN, these miRNAs like miR-142-5p and miR-24 can activate the PI3K/AKT pathway, which promotes OC cell growth and invasion. Additionally, miRNAs like miR-655 might act as a tumor suppressor. miR-655 appears to block the PTEN/AKT pathway, hindering OSCC cell proliferation and invasion [[93], [94], [95], [96]].

Furthermore, elevated levels of miR-155 observed in OSCC patients and bone marrow-derived mesenchymal stem cells (BMSCs) with increased miR-155 expression may foster OSCC proliferation and dissemination by suppressing PTEN [85,97]. A single miRNA can have opposing functions depending on the context. For instance, the miR-200 family hinders cancer spread but might also make the surrounding tissue more receptive to tumor invasion [98,99]. Table 2 highlights the complex role of miRNAs in OSCC progression.

a. lncRNA in OC

**Table 2:** The findings from numerous research on the expression of miRNAs in biological samples from OSCC vs healthy controls.

miRNA	Sample Source	Impact of miRNA on OC	Reference
(miR-222-3p, miR-150-5p, and miR-423-5p) arying levels between healthy and cancer groups, (miR-130b-3p and miR-221-3p) for comparison	Two hundred and fifty patients (70 normal, 66 Oral leukoplakia, and 114 OSCC)	miR-222-3p and miR-423-5p connected to more advanced cancer stages. (miR-150-5p/miR-222-3p and miR-150-5p/miR-423-5p) effectively differentiates between healthy and precancerous conditions.	[100]
miR-24-3p, miR-21-5p, let-7c-5p, miR-99a-5p, miR-100-5p	Patients (total n = 190), including OSCC (n = 53) and oral potentially malignant disorders (OPMDs) (n = 74)	86.8% sensitivity and 81.5% specificity. Older age and female gender are associated with higher dysregulation score (dSCORE). OPMDs subgroups have significantly different dSCOREs.	[101]
miR-26a	The KB human OC cell line	increases miR-26a expression, inhibiting proliferation and promoting apoptosis,	[102]
miR-486-3p	OSCC patients Tissue, saliva, and plasma samples (n = 46)	Promoting gene *ANK1* methylation leads to downregulation of miR-486-3p, which inhibits gene expression of *DDR1*, promoting cancer growth	[103]
miR-24-3p	49 patients with OSCC	Salivary exosomal miR-24-3p is a promising diagnostic biomarker for OSCC, targeting *PER1* to maintain cell proliferation	[104]
Five miRNAs up-regulated in OSCC patients (miR-412-3p, miR-489-3p, miR-512-3p, miR-597-5p, and miR-603) and eight miRNAs expressed only by OSCC patients (miR-27a-3p, miR-302b-3p, miR-337-5p, miR-373-3p, miR-494-3p, miR-517b, and miR-520d-3p, miR-645)	A total of 21 patients with OSCC (12 men and 9 women)	Two miRNAs (miR412-3p and miR-512-3p) were found to be overexpressed in OSCC patients, while two miRNAs (miR-302b-3p and miR-517b3p) were selectively enriched in EVs from OSCC patients	[105]
miR-375	The CAL-27, Tca8113, UM1 and UM2 OSCC cell lines	MiR-375 expression levels were reduced in metastatic OSCC cell lines UM1 and CAL-27, and overexpression suppressed migration and invasion. PDGF-A was identified as a direct target gene of miR-375, and overexpression reversed its effect on migration and invasion in *UM1* cells.	[106]
miR-203	Normal human oral keratinocytes (NHOKs), The human OC cell line YD-38	MiR-203 expression in YD-38 cells was found to be down-regulated, decreasing cell viability and increasing DNA segmentation. Over-expression of miR-203 led to apoptosis in YD-38 cells. The down-regulation of Bmi-1, an oncogene, was also observed, with both mRNA and protein levels reduced	[107]
miR‐211	OSCC tissues and cell lines (SCC6, SCC9, SCC25, HN4, and HN6)	BIN1 and miR-211 were low in OSCC tissues and cell lines, inhibiting their proliferation, migration, and invasion. MiR-211, a highly expressed miRNA, could bind with BIN1's 3′-UTR, triggering its degradation. MiR-211 inhibitors could suppress malignant behaviors by upregulating *BIN1* expression and inhibiting the EGFR/MAPK pathway	[108]
miR-5580-3p	40 patients diagnosed with OC , The human OC cell lines (SCC-4, SCC-9 and Cal-27), as well as the human oral epithelial cell line (HOEC; cat. no. BNCC340217)	miR-5580-3p was downregulated while LAMC2 was upregulated in OC tissues, and miR-5580-3p suppressed OC cell growth and motility by inhibiting *LAMC2*	[109]
hsa-miR-204-5p ,hsa-miR-375, hsa-miR-4497 ,hsa-miR-1291, hsa-miR-4492, hsa-miR-3196 , hsa-miR-6087, hsa-miR-4508, hsa-miR-4485-3p, hsa-miR-3195, hsa-miR-3687, hsa-miR-3648, hsa-miR-6510-3p, hsa-miR-4516, hsa-miR-7704 ,hsa-miR-3656, hsa-let-7c-5p, hsa-miR-4532, hsa-miR-4488, hsa-miR-99a-3p, hsa-miR-125b-5p, hsa-miR-139-5p, hsa-miR-3651, hsa-miR-125b-2-3p, hsa-miR-99a-5p ,hsa-miR-31-3p ,hsa-miR-424-5p , hsa-miR-196b-5p, hsa-miR-877-5p, hsa-miR-7-5p, hsa-miR-135b-5p, hsa-miR-31-5p , hsa-miR-142-3p, hsa-miR-187-3p, hsa-miR-19a-3p, hsa-miR-708-3p, hsa-miR-223-3p, hsa-miR-32-5p, hsa-miR-18a-5p, hsa-miR-301a-3p, hsa-let-7a-3p, hsa-miR-21-5p, hsa-miR-455-5p hsa-miR-92b-3p, hsa-miR-21-3p, hsa-miR-142-5p, hsa-miR-944, hsa-miR-20a-5p	fve patients diagnosed with OSCC of the oral cavity, In total, 255 distinct miRNAs were detected in tissue samples, while 381 unique miRNAs were identified in serum samples.	hsa-miR-21-5p is up-regulated in cancerous tissue and is considered an oncogene. hsa-miR-375 was down-regulated in cancerous tissue and is associated with cancer aggressiveness. hsa-miR-31-3p is up-regulated in cancerous tissue and is associated with decreased survival. hsa-miR-99a-5p, hsa-let-7a-3p and hsa-miR-32-5p can be good candidates as markers for non-invasive diagnosis of patients with OSCC	[110]
let-7a, let-7d, let-7f and miR-16 , miR-29b, miR-142-3p, miR-144, miR-203, and miR-223, f miR-1275	discovery cohort (n = 29) and validation cohort (n = 61) of primary OSCC tissue specimens	let-7a, let-7d, let-7f, and miR-16 were downregulated, while miR-29b, miR-142-3p, miR-144, miR-203, and miR-223 were upregulated. Notably, miR-1275 exhibited variable expression associated with lymph node invasion, and miR-223 correlated with advanced tumor stage/size. In silico analysis highlighted the involvement of these miRNAs in modulating tumor suppressor and oncogene pathways, suggesting a dual role in PI3K/Akt and p53 signaling regulation.	[111]
miR-376c-3p	49 paired OSCC and normal oral epithelial tissue	MiR-376c-3p targets *HOXB7* to decrease OSCC fission, proliferation, migration, and invasion while inducing cell death	[112]
miRNA-124	saliva of 25 patients with oral squamous cell carcinoma	Lower expression in the saliva of patients with oral squamous cell carcinoma compared to healthy individuals	[113]

LncRNAs are transcripts that originate from regions between genes or overlapping existing genes in either a sense or antisense direction. They often have a structure similar to mRNA with a cap at the 5' end, a polyA tail at the 3' end, and splicing within the sequence. Interestingly, lncRNAs can be found within a single cell in the nucleus, cytoplasm, or both [[114], [115], [116]]. The location of a lncRNA determines its function within the cell. Nuclear lncRNAs, the most common type, play a regulatory role in nuclear structure, chromatin organization, and epigenetic modifications. They can even interact with DNA to form R-loops, influencing gene transcription and maintaining genomic stability.

**Figure 2:**
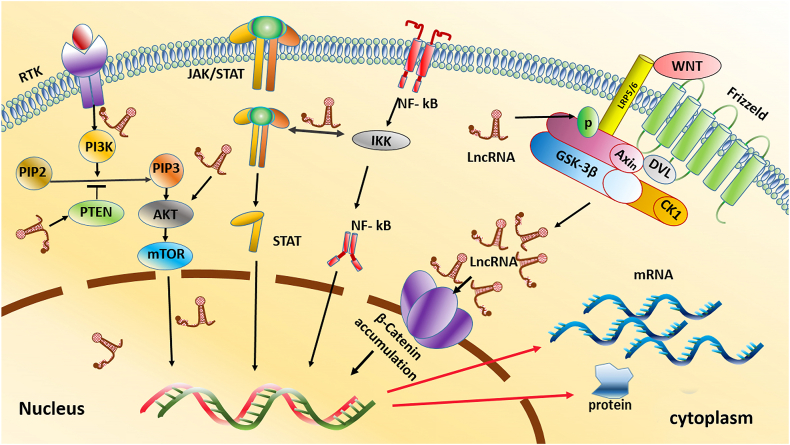
The role of lncRNAs in distinct signaling pathways involved in OC development and progression.

Meanwhile, cytoplasmic lncRNAs take charge of protein production by influencing mRNA stability and translation. They also interact with proteins and miRNAs, adding another layer of complexity to cellular regulation [[117], [118], [119], [120]]. LncRNAs influence gene regulation through chromatin remodeling, transcription activation, RNA interference, and splicing. Interestingly, most lncRNAs are upregulated in OC tissues, while a few, including C5orf66-AS1, CASC2, ENST00000470447.1, FALEC, LINC01315, and MORT, are downregulated [[121], [122], [123], [124], [125], [126], [127], [128]]. Despite the complexity of chromatin remodeling, where histone modifications play a crucial role in gene regulation, some lncRNAs like HOTAIR and FALEC have been identified to influence histone modification and gene expression in OSCC [126,129]. Recent research suggests that lncRNAs can influence gene expression by altering this chromatin architecture or even kicking out architectural proteins like *CTCF*, which plays a crucial role in chromatin structure, potentially disrupting the loops and gene regulation [130]. LncRNAs influence OSCC development by interacting with DNA or proteins in the nucleus. For instance, lncRNA lnc-p23154 blocks miR-378a-3p, increasing *Glut1* expression and fueling glycolysis, and lncRNA HAS2-AS1 activates *HAS2*, promoting hypoxia-induced cancer progression in OSCC cells [131,132]. Researchers believe lncRNAs also play a role in OSCC metastasis. These lncRNAs could influence migration, invasion, and the formation of new lymph vessels (lymphangiogenesis) through various pathways, potentially impacting whether OSCC spreads. Notably, epithelial-to-mesenchymal transition (EMT) is crucial for OSCC metastasis, and lncRNAs appear to be involved in regulating EMT in these cancer cells [133,134]. Additionally, studies have identified hypoxia-induced lncRNAs like LncHIFCAR that directly interact with a protein called HIF-1α and help HIF-1α to activate genes involved in metastasis, further promoting OSCC progression [135,136]. OC research reveals genetically altered pathways, including the Wnt/β-catenin pathway and the PI3K/AKT/mTOR pathway, which are crucial for cell development and regeneration and are often hyperactivated. LncRNAs are crucial in regulating these pathways in the context of OC [[137], [138], [139], [140], [141]] (figure. 2). LncRNAs can either affect genes epigenetically (e.g., *FALEC* recruiting *EZH2* to methylate specific locations and condense chromatin) or directly influence transcription (e.g., by inhibiting NF-κB transcription factors and suppressing Twist expression) [129,142,143]. LncRNAs can also affect signaling pathways, such as inhibiting the Hippo pathway by interacting with *LATS1* protein to inhibit the Hippo pathway, reducing YAP1 phosphorylation. Additionally, lncRNAs can act as miRNA sponges, like H19, soaking up miR-138 to increase its target genes' expression [144]. Finally, lncRNAs can stabilize mRNAs, like binding to CEBPA mRNA, to enhance its stability and increase its target transcripts [145]. Further investigation into lncRNAs holds promise for developing novel diagnostic and therapeutic strategies for OC. By understanding how these molecules regulate gene expression and cellular processes, researchers may be able to identify new targets for intervention and improve patient outcomes. Several lncRNAs and their functions in OC are presented in the table 3.

**Table 3:** The impact of various lncRNAs on OC

lncRNA	Sample Source	Impact of lncRNA on OC	Reference
lncRNA MALAT1	OSCC cell lines (HSC3, SCC9, SCC15 and SCC25) and (NHOK) cells-OSCC tissues (n=20 , 16 male and 4 female)	Upregulation of MALAT1 promotes proliferation and invasion of OSCC cells by targeting the miR-101/EZH2 axis.	[146]
lncRNA GAS5	OSCC tissues samples (n = 6), cell lines, including HSC3, HSC6, SCC15, SCC25, cal-27, and UM1	GAS5 regulates the miR-21/PTEN axis, controlling cell proliferation, migration, invasion, and EMT in OSCC, suggesting targeting GAS5 or the miR-21/PTEN axis could be lead to novel OSCC therapies.	[147]
lncRNA MEG3	The OSCC cell lines SAS (tongue squamous cell carcinoma) and GNM (gingival carcinoma neck metastasis)	correlation between low MEG3 expression, increased miR-421 activity, and enhanced OC stem cell (CSC) features	[148]
lncRNA CASC2	OSCC tissues (n=69) , OSCC cell lines (Tca8113, SCC-9, TSCCa, CAL-27) an normal oral keratinocyte (NOK) cell line	CASC2 expression is downregulated in OSCC tissues and cells, causing poor clinical outcomes. Overexpression inhibits cell proliferation and may compete with miR-21, regulating PDCD4 expression.	[124]
lncRNA CASC9	OSCC tissues (n=35) ,OSCC cells, SCC15 and CAL27 cells	lncRNA CASC9 is highly expressed in OC and promotes tumor growth. CASC9 autophagy and apoptosis through the AKT/mTOR pathway.	[149]
lncRNA XIST	TSCC samples from patients (n = 3), CAL27 and SCC9 cells	regulating XIST and miR-29b expression, which might influence p53 and lead to inhibited cell growth, invasion, and increased apoptosis in TSCC	[150]
lncRNA GACAT1	TSCC samples from (n = 25), Tca8113, SCC1, SCC-4 and SCC-15 TSCC cells	GACAT1 overexpression in TSCC, potentially linked to decreased miR-149 expression	[151]
lncRNA MORT	OSCC samples from patients (n = 59), Cells of SCC090 and SCC25 cell lines	Positive correlation between ROCK1 and cancer progression, negative correlation between MORT and cancer progression. MORT overexpression inhibits proliferation by downregulating *ROCK1*	[128]
lncRNA HOXC13-AS	OSCC samples from patients (n = 56), OSCC cells (Fadu, TSCCA, HSU3, SCC15)	HOXC13-AS upregulation promotes OSCC cell proliferation, migration, and EMT *via* miR-378g/HOXC13 axis	[152]
lncRNA FENDRR	OSCC samples from patients (n = 7), OSCC cell lines SCC25 and CAL27	FENDRR, a lncRNA downregulated in OSCC, inhibits angiogenesis by suppressing the PI3K/AKT pathway in CAFs	[153]
lncRNA PAPAS	OSCC samples from patients (n = 68) and 52 healthy volunteers, SCC090 and SCC25 OSCC cell lines	lncRNA PAPAS promotes OSCC progression by upregulating TGF-β1, making PAPAS a potential therapeutic target	[154]
lncRNA IFITM4P	Microarray: NM (n = 3), OL (n = 4), and OSCC (n = 5) samples, Leuk-1 (OL) and HN4 (OSCC), qRT-PCR validation: NM (n = 23), OL (n = 67), and OSCC (n = 46)	Scaffolding interaction between *SASH1* and *TAK1*, leading to increased NF-κB and PD-L1 expression, and immune escape of OL cells	[155]
lncRNA TIRY	OSCC samples from patients (n = 145) , TCA8113 cell line	TIRY acts as a miRNA sponge, downregulating miR-14 and leading to increased expression of EMT markers (Snail, FOXC2, a-SMA, b-catenin, FSP1) *via* Wnt/β-catenin signaling, promoting invasion and metastasis of OSCC cells	[156]
lncRNA MINCR	OSCC tissues and Human OSCC cell lines (TSCCA, Tca8113, SCC25)	MINCR, previously thought to suppress tumors, may act as an oncogene in OSCC by promoting cell proliferation, invasion, and the Wnt/β-catenin signaling pathway	[157]
lncRNA MIR4435-2HG	OSCC tissues samples (n = 10) and normal oral tissues (n = 10), Human immortalized oral epithelial cells, SCC-9, and HSC-4 cells	lncRNA MIR4435-2HG: Upregulated by F. nucleatum, suppresses miR-296-5p, miR-296-5p: Downregulated by F. nucleatum, inhibits SNAI1, SNAI1: Promotes EMT, Akt2: Downstream of miR-296-5p, inhibits SNAI1.	[158]
lncRNA PARROT, MYCNUT, DANCER, KTN1-AS1	OSCC tissues samples (n = 30) and normal oral tissues (n = 30)	Overexpression of lncRNAs PARROT, MYCNUT, DANCR, and KTN1-AS1 in OSCC tumors may serve as useful biomarkers for OSCC detection, with high accuracy in MYCNUT and KTN1-AS1 levels in larger tumors	[159]
LncRNA TUG1	oral epithelial cell line NOK and OSCC cell lines SCC‐4, SCC‐9, SCC‐25, CAL‐27, HN‐6, and TCA8113	LncRNA TUG1, promotes OC cell growth and migration by competing with miR-524-5p, which reduces a gene *DLX1* causing cancer development.	[160]
lncRNA 1 (CILA1)	CAL27-res and SCC9-res cells in TSCC	CILA1 fuels cisplatin resistance and EMT in TSCC. blocking CILA1 makes cancer cells more sensitive to cisplatin and reverses EMT. CILA1 achieves this through the Wnt/b-catenin pathway	[161]
lncRNA FOXD2- AS1	24 cases of fresh laryngeal squamous cancer tissues and adjacent normal tissues, Human LSCC cell line Hep2 , and the TU-212 cell line	FOXD2-AS1 promotes resistance to chemotherapy in laryngeal squamous cell carcinoma (LSCC) by increasing cancer stemness. FOXD2-AS1 could be a target for new therapies to improve chemotherapy	[162]
lncRNA (LINC00958)	cell lines human oral keratinocyte (HOK), TSCC (A-253), OSCC cell (HSC-4), TSCC (CAL-27) and TSCC (SCC-4)	LINC00958 is elevated in OC cells and promotes their survival. It reduces miR-4306 that targets an inflammatory gene, *AIM2*, and interacts with *SIRT1*, a protein that regulates cell death and reduces p53 levels.	[163]
LncRNA AC007271.3	OSCC tissues samples (n = 97), Normal human oral epithelial cell line (HOK) and oral squamous cell lines (SCC9, SCC15 and SCC25)	AC007271.3 promotes cancer cell growth, migration, and reduces cell death by activating the Wnt/β-catenin signaling pathway. AC007271.3 downstream targets CyclinD1, c-myc, and Bcl-2.	[164]
LncRNA HOTAIR	OSCC tissues samples (n = 44), cell line (HOK), HN4 and HN6, SCC9, Cal27	HOTAIR negatively correlates with miR-326 expression and acts as a competitive endogenous RNA to regulate metastasis-associated gene 2 (*MTA2*). High *MTA2* expression is linked to advanced OSCC and poor prognosis. HOTAIR-miR-326-*MTA2* axis plays a crucial role in OSCC progression	[165]
LncRNA FOXD2-AS1	Cal27 and SCC9 cells	FOXD2-AS1 was significantly upregulated in Cal27 and SCC9 cells & FOXD2-AS1 enhances OSCC malignant cell behaviors by interacting with miR-378 g to regulate CRABP2 expression.	[166]
lncRNAs TMPO-AS1 lncRNAs DDX11-AS1	100 HNSCC tumors and normal adjacent tumors	DDX11-AS1 expression was also significantly higher in HNSCC samples than in NATs Similarly, TMPO-AS1 levels were significantly elevated in HNSCC samples compared to NATs.	[167]
LINC00342	CAL-27 and FaDu cell lines	upregulated in OC cells	[168]
LncRNA NR2F2-AS1	OSCC Cell lines	NR2F2-AS1 overexpression has inhibitory effects on OSCC cell EMT, migration, invasion, and angiogenesis as well as tumor growth and metastasis.	[169]

### 3.2 CircRNAs in OC

**Figure 3:**
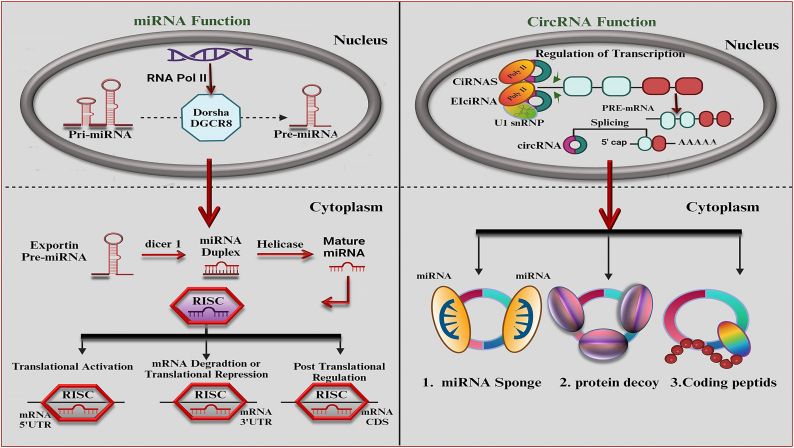
MiRNAs are essential RNA molecules in gene regulation, synthesized through a multi-step process. They begin with the transcription of a primary miRNA, then processing into a precursor and mature miRNA, affecting gene expression. CircRNAs interact with Pol II machinery to promote host gene transcription, while EIciRNAs induce transcription through U1 snRNP and Pol II. They can compete with back-splicing and linear splicing, serve as sponges for the miRNA-Ago2 complex, regulate proteins, and be translatable RNAs.

CircRNAs have recently become a hot topic in OC research. Recent research shows that circRNAs are formed through a different process (back-splicing) compared to linear RNAs (conventional splicing) [170]. There are two main models for circRNA formation: intron-pairing-driven and lariat-driven, and the specific process depends on the type of circRNA (exonic circRNAs (ecircRNAs), exon-intron circRNAs (EIciRNAs), and circular intronic RNAs (ciRNAs)). Some RNA-binding proteins (RBPs) can regulate circRNA synthesis. The exact mechanism is still unclear, but circRNAs are widely found in organisms and are very stable due to their closed-loop structure [[171], [172], [173], [174], [175], [176], [177]]. CircRNAs can act like sponges for RBPs, affecting protein levels in the cell. For instance, circ-MBL binds to MBL proteins, which are essential for various cellular functions [178,179]. These findings highlight a broader role for circRNAs in interacting with proteins and influencing cellular behavior. In normal mRNA, ribosomes start translation by scanning from the beginning (5' cap) to find the starting signal (initiation codon). CircRNAs lack the cap and cannot be translated this way. However, circRNAs can still be translated using two main mechanisms: 1) m6A-mediated translation and 2) internal ribosome entry site (IRES)-dependent translation. IRES allows ribosomes to attach directly to the circRNA and initiate translation. In some cases, both mechanisms work together to improve translation efficiency [[180], [181], [182], [183], [184], [185], [186]] (figure. 3). CircRNAs initially formed as mRNA precursors but ultimately became ncRNAs and are thought to indirectly influence gene expression by acting like miRNA sponges. They bind to miRNAs, preventing them from silencing their target genes. CircRNAs are sometimes called miRNA sponges [176,[187], [188], [189]]. CircRNAs can act as either tumor promoters or suppressors. Some, like circ_0002185 and circ_PVT1, are upregulated in OC and promote cancer cell growth. Others, like circ_PKD2, are downregulated and inhibit cancer cell growth. CircRNAs influence OC by affecting critical cellular pathways like Notch, VEGF, MAPK, and PI3K/AKT [[190], [191], [192], [193], [194], [195], [196]]. The study shows circCDR1as promotes the survival of OSCC cells under low oxygen conditions by encouraging autophagy, a cellular recycling process. CircCDR1as boosted hypoxia-mediated autophagy by targeting key autophagy regulators, inhibiting mTOR activity, and upregulating the AKT and ERK1/2 pathways [193]. Studies have identified OSCC-specific circular RNAs in saliva, notably has-circ-0001874 and has-circ-0001971, which are significantly elevated in OSCC patients, making them potentially valuable tools for diagnosing and monitoring the disease. These unique circRNAs might also serve as therapeutic targets. Scientists may be able to prevent cancer cell development by utilizing siRNA to interfere with circRNA expression. For instance, targeting circRNA-100290 with siRNA has inhibited OSCC cell proliferation in lab studies and animal models [[197], [198], [199]]. In conclusion, circRNAs have emerged as a significant focus in OC research due to their unique formation process, stability, and regulatory functions. Table 4 highlights several circRNAs in biological samples related to OSCC cell progression.

**Table 4:** Some studies on the expression of circRNA in biological samples related to OSCC.

Circ RNA	Sample Source	Impact of circ RNA on OC	Reference
Has-circ_000334, Has-circ_006740, Has-circ_006371	42 oral squamous cell carcinoma patients. OSCC cell lines (SCC9, SCC15, SCC25, CAL27) and HOK (normal oral keratinocyte cell line).	The OSCC tissues have significantly increased levels of circ_000334, circ_006740, and circ_006371 in comparison to normal tissues.	[200]
Has-Circ-DOCK1	The human OSCC cell lines CAL-27, SCC-9, SCC-25 and HOK. oral carcinoma tissues and adjacent normal tissues	circDOCK1 has an apoptotic regulatory role and is increased in OSCC. In OSCC tissue and cell lines, BIRC3 is substantially expressed while miR-196a-5p is expressed at a low level. Apoptosis of OSCC cells can be controlled by the circDOCK1/miR-196a-5p/BIRC3 pathway.	[201]
Hsa_circ_0008309	45 pairs of frozen OSCC tissues and adjacent normal tissues. SCC-15 and CAL27 cell lines.	In OSCC tissues, hsa_circ_0008309 has been markedly downregulated. In SCC15 and CAL27 cell lines, hsa_circ_0008309 is overexpressed, miR-136-5P and miR-382-5P expression is suppressed, and ATXN1_8300h protein levels are elevated. In OSCC cell lines, hsa_circ_0008309 may control the hsa_circ_0008309-miR-136-5P/miR-382-5P-ATXN1 pathway.	[202]
Hsa_circ_001242	40 OC tissue samples and paired adjacent normal tissues. OSCC cell lines (SCC-9, SCC-15, SCC25, CAL-27) and human normal oral keratinocyte (HOK)	The expression of hsa_circ_001242 was downregulated in OSCC cell lines and tissues. There exists a negative correlation between the expression of hsa_circ_001242, tumour size, and T stage.	[203]
Has-Circ_0014359	30 pairs of tumor tissues and the adjacent normal tissues. Human OSCC cell lines (PE/CA-PJ41, SCC15, SCC25, and HSC-4) and oral mucosa epithelium cell line (NHOK)	Comparing OSCC tissues to normal tissues, there is a considerable increase in the expression of circ_0014359. When compared to adjacent normal tissues, the expression of miR-149 in tumour tissues is markedly lower. The upregulation of miR-149 in OSCC cells following circ_0014359 knockdown suggests that miR-149 is circ_0014359's target.	[204]
Has-Circ-MMP9	74 paired OSCC and corresponding non-tumor normal tissues. plasma samples from 16 healthy control and 25 OSCC patients. OSCC cell lines including HN4, UM1, SCC-9, SCC-15, HSC-3, and CAL-27 and the normal oral keratinocyte (NOK) cells.	In OSCC cells, circ-MMP 9 can suppress miR-149. The expression of circ-MMP9 is markedly elevated in the tissues, plasma, and cell lines of OSCC. In order to prevent MMP9 mRNA from being degraded, circ-MMP9 can physically attach to AUF1 and miR-149. This interaction increases MMP9 expression and promotes OSCC invasion and metastasis. One positive regulator of OSCC metastasis is circ-MMP9.	[205]
Has-circ-PVT1	115 HNSCC patients CAL27, FaDu, A253, and H1299 cell lines	Tumor samples have an upregulation of circPVT1. showed the only patients with TP53 mutations had significantly higher levels of circPVT1, demonstrating the association between mut-p53 and circPVT1 in HNSCC. that circPVT1 acts as an oncogene in HNSCC and that its expression is transcriptionally regulated by the mut-p53/YAP/TEAD complex.	[206]
Hsa_circ_0001874 and hsa_circ_0001971	90 OSCC patients, 70 OLK subjects	When comparing OSCC patients to OLK patients, there was a significant rise in the expression of hsa_circ 0001874, as well as hsa_circ 0001971.	[207]
Has-Circ_0049396	27 pairs of OSCC and adjacent tissues. The OSCC cell lines (HSC-2, SJG-1, and H157), HEK293T cells, and human immortalized oral epithelial cells (HIOEC)	Through control of the miR-663b/ENDOU axis, overexpression of circ_0049396 reduces the malignant behavior of OSCC cells. The OSCC cells exhibited a considerable increase in miR-663b levels. Circ_0049396 targets miR-663b in OSCC cells. By modifying the miR-663b/ENDOU axis, circ_0049396 overexpression can suppress the malignant cell behaviour of OSCC and decrease tumour growth in vivo.	[208]
Hsa_circ_0112879	42 OSCC patients. The human OSCC cell lines SCC9, SCC15, SCC25, CAL27 and the human oral keratinocyte (HOK) cell line.	expression of hsa_circ_0112879 has down-regulated in OSCC tissues and cell lines. Based on bioinformatics analysis, has_circ_0112879 may have strong interactions with hsa-miR-654-3p, hsa-miR-338-3p, and hsa-miR-155-3p.	[209]
Hsa_circ_0086414	Tumor tissues and AHTs were obtained from 55 patients. CAL27 cells, SCC25 cells and Normal (HaCaT) cells	In OSCC cell lines and tissues, there was a significant decrease in the expression of hsa_circ_0086414. Additionally, individuals that exhibit hsa_circ_0086414 less frequently have higher TNM stages and lymph node metastases that are positive.	[210]
Hsa_circ_0033144 (CircBCL11B)	50 patients diagnosed with OSCC. OSCC cell lines including Cal-27, FADU, OECM1, SAS, HSC3 and Normal oral keratinocytes (NHOK)	circBCL11B and miR-579 work together to control the amount of LASP1 expression.In OSCC tissues and cell lines, miR-579 is downregulated, which could be related to circBCL11B overexpression.	[211]
hsa-circ-0006203 hsa-circ-0004872	OSCC tissues	Both them are downregulated in OSCC	[212]

### 3.3 snoRNA in OC

**Figure 4:**
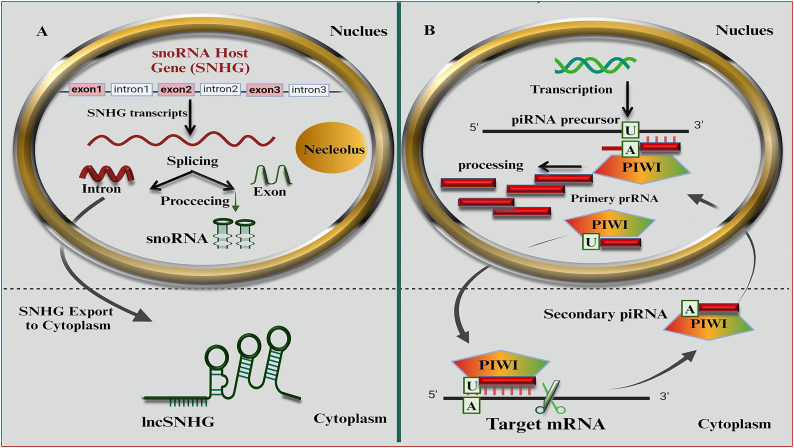
**A.** SNHGs are protein-coding and non-protein-coding genes transcribed into primary SNHG transcripts. These transcripts are cut into exons and introns, re-spliced, and translocated into the cytoplasm. Intronic sequences are processed into mature snoRNA assembled into snoRNPs **B**. The piRNA/PIWI complex is a ribonucleoprotein complex responsible for genome integrity through transposable element silencing in the germline. Primary piRNAs have uridine bias, while secondary piRNAs have adenosine bias. The complex is active in epigenetic silencing, epigenetic activation, and mRNA degradation in the nucleus and cytoplasm. It recruits heterochromatin protein 1 and histone methyltransferases and is involved in epigenetic activation.

Small nucleolar RNAs (snoRNAs) exist in the C/D box and H/ACA box. C/D snoRNAs, armed with their distinctive C and D box sequences, target specific sites on ribosomal RNA (rRNA) for a chemical modification called 2'-O-methylation. H/ACA snoRNAs, identified by their H and ACA boxes, specialize in another rRNA modification, pseudo-uridylation. Interestingly, some snoRNAs can be further processed into microRNAs that silence protein production in the cytoplasm or even into piwi-interacting RNAs that control the movement of transposable elements within the nucleus [[213], [214], [215]]. Some snoRNA genes have both introns and exons, but only the intronic regions are used to create snoRNAs. If the entire transcript, including exons, is retained, it functions as a small nucleolar RNA host gene (SNHG). The SNHG's internal structure and availability influence the snoRNA's level within the cell [213,216] (figure. 4). SnoRNAs were recently discovered to be involved in OC development. They can promote cancer cell growth, migration, and invasion by affecting various pathways. Some snoRNAs are upregulated in OC patients and might be valuable tools for diagnosis and therapy. For instance, SNHG3 promotes cancer progression and correlates with poor patient survival, suggesting its potential as a prognostic marker [[217], [218], [219], [220], [221], [222]]. SNHG molecules, located within cells, can influence gene activity by altering DNA methylation or interacting with transcription-controlling proteins. They can act as sponges in the cytoplasm, absorb microRNAs, or prevent protein breakdown through interaction or complex formation [223]. Several studies highlight the potential of snoRNAs as biomarkers for OC (table 5). Analysis of large datasets revealed snoRNAs associated with crucial pathways in cancer progression, including cell proliferation, invasion, and metastasis.

**Table 5:** The impact of various ncRNAs, including snoRNAs and piRNAs, on OC

SnoRNA/Pi RNA	Name	Sample Source	Impact of Non-coding RNA on OC	Reference
SnoRNA	SNORD114‐17‐NNMT SNORA36B‐CIRBP U3(chr2)‐CTNNBL1 U3(chr17)‐ZNF101 SNORA36B	510 HNSCC samples	SNORA36B and U3 (chr17), two of the five snoRNAs, are protective, whereas the other three are risk factors.	[225]
SnoRNA	14qI-4, 14qII-22, ACA17, ENSG00000263442, ENSG00000264591, ENSG00000265325, ENSG00000265607, ENSG00000266646, ENSG00000266755, U84, mgh18S-121, U18A, U8, ENSG00000207118, 14qII-12, U28	8 OSCC samples and eight control samples of keratinized gums	15 were overexpressed, whereas one was under expressed. Patients with ENSG000002645191, ENSG00000263442, ENSG00000265325, ENSG00000265607, ENSG00000266646, ENSG00000266755, ENSG00000207118, U8, and U28 expression levels lower than the mean had a longer survival time. There are differences in 14qII-22 expression levels between males and females, as well as lower U28 expression levels in cases with strong differentiation.	[233]
SnoRNA	SNHG20	90 patients with OSCC The NHOK, TSCCA, SCC15, SCC25,and CAL27 cell lines	It was observed that TSCCA, SCC15, SCC25, and CAL27 cells expressed more SNHG20. In OSCC tissues, SNHG20 was markedly elevated in comparison to nearby non-tumor tissues. SNHG20 modulates the miR-19b-3p/RAB14 axis to inhibit the biological activity of OSCC.	[234]
SnoRNA	SNHG16	29 pairs of OSCC tissues and normal tissues. SCC-25, CAL-27, Tca8113 and TSCCA cells and normal human oral keratinocyte (NHOK)	When comparing normal tissues and human oral keratinocyte cells to OSCC tissues and cell lines, SNHG16 was overexpressed. In OSCC tissues, there was an obvious positive connection between SNHG16 and c-Myc expression.	[235]
SnoRNA	SNHG15	Human OSCC cell lines (SCC-15, SCC-9, SCC-25, and HSC-2) and normal oral keratinocytes (NOK)	In OSCC cells, SNHG15 is overexpressed. The ability of OSCC cells to proliferate, migrate, and invade was inhibited by SNHG15 inhibition, although this also caused apoptosis in these cells. SNHG15 elevated DAAM1manifestation through the use of miR-188-5p, contributing to the OSCC process.	[221]
piRNA	piR-33422	HOK cells (GEO database accession numbers: GSE196674 and GSE196688) in TSCC	down-regulated piR-33422 might increase the expression of a gene (FDFT1) involved in cancer progression and Down-regulated: OGA, BDH1, TAT, HYAL4	[159]
piRNA	PIWIL2	human 137 OSCC, THP-1 cells	PIWIL2 expression is linked to worse outcomes in OSCC and a higher risk of developing OSCC from precancerous lesions (OL). PIWIL2 is present in both cancer cells and immune cells in the tumor environment	[236]
piRNA	PIWIL2	60 OSCC patients, cell lines (Tca8113, SCC9, SCC25, CAL27, HN12, HSU3, and FADU) and a normal human oral keratinocyte (NHOK) cell line	Silencing PIWIL2 in the Tca8113 OSCC cell line induces cell cycle arrest and apoptosis and significantly suppresses migration and invasion abilities, correlated with the malignancy stage of OSCC.	[237]
piRNA	piR-1037	SCC4, SCC9, SCC15 and SCC25 OSCC cell lines or tumor xenografts	Suppressing piR-1037 upregulation increases the sensitivity of OSCC cells to CDDP and inhibits cell motility by affecting EMT. piR-1037 directly binds to XIAP, a key apoptotic inhibitor involved in chemoresistance	[238]

Interestingly, the levels of the top five snoRNAs (SNORD114-17: ENSG00000201569, SNORA36B: ENSG00000222370, SNORD78: ENSG00000212378, U3: ENSG00000212182, and U3: ENSG00000212195) were linked to patient survival, suggesting their importance in disease development and prognosis in OC [[224], [225], [226]]. Further research is crucial to understanding how snoRNAs influence OC development fully. However, the evidence strongly suggests that snoRNAs hold significant promise for improving OC diagnosis and potentially even treatment strategies in the future.

### 3.4 piRNA/PIWI in OC

Further research indicates that piRNA is extensively expressed in somatic cells and plays a crucial role in various essential functions, including regulating the cell cycle, cell proliferation, energy metabolism, and the immune microenvironment. The regulatory function of piRNAs are achieved by influencing gene expression through epigenetic modifications and regulation of various signaling pathways [[227], [228], [229]]. The association between piRNAs and PIWI proteins inhibits transposable element (TE) activity and regulates encoded mRNAs *via* several mechanisms [230]. Researchers identified piRNAs (piR354, piR415, piR832, and piR1584) that can interact with mRNA molecules in the OC mice model. Interestingly, genes like GALNT6, SPEDF, and MYBL2 seem to be involved with these piRNAs and may play a role in stopping or promoting the growth of specific head and neck tumors [231]. The presence of low levels of piR-58510 and piR35373 is linked to extended survival among patients with head and neck cancer [232]. Further research on piRNAs could lead to novel diagnostic tools and therapeutic targets for OC.

## 4 The pathways involved in OC are an essential issue in disease identification

Cancer is a group of diseases that induce aberrant cell development as a result of changes in the way different genes are expressed. The molecular network is altered, and signaling cascades are changed due to these gene modifications. These alterations ultimately result in cellular dysfunction, which is followed by systemic failure that ends in death [239]. Three significant pathways—the "cell cycle pathway," the "endocytosis pathway," and the "pathways in cancer"—were among the dozens that are aberrantly active in cancer. Angiogenesis, cell proliferation, and cell survival rely on the "cell cycle pathway." The G1 and G2 phases are the temporal gaps between DNA replication and mitosis, allowing mitotic cells to proceed through their cell cycle. The genes encoding cyclin-dependent kinases, p53, and retinoblastoma protein (Rb), are among the several genes in this pathway linked to cancers [[240], [241], [242], [243], [244], [245], [246]].

More research is necessary to fully understand the mechanisms underlying endocytosis in OSCC, even though it has been identified as a critical pathway. Endocytosis transports ligands, nutrients, lipids, and proteins from the plasma membrane into the core of cells and removes them from the cell surface. Growth factor receptor oncogenic mutants show defective endocytosis; pharmaceutical medicines may use this distinct endocytic system in cancer to treat patients effectively [247,248].

Wnt, Ras, p53, and cell death are only a few of the tumor-related genes and signaling pathways involved in cancer pathways. Tumor metastasis and carcinogenesis are closely linked to abnormal Wnt pathway activation. Ras is a molecular switch for signal transduction that starts in the cell membrane. It is a tiny G protein that cycles between inactive GDP-bound and active GTP-bound forms. Cancer cells can avoid apoptosis by deregulating apoptotic signaling and activating anti-apoptotic systems, resulting in uncontrollably proliferating cells, tumor growth, resistance to treatment, and cancer recurrence [[249], [250], [251], [252]]. Some of these signaling pathways and the factors affecting them are summarized in the table 6.

**Table 6:** Some research studying the role of non-coding RNAs in various pathways implicated in OC.

Pathway	Method	samples	Result	Reference
miRNA-485-5p/KRT17/integrin/FAK/Src/ERK/β-catenin	Microarray analysis & Plasmid construction & Western blot assay & RT-qPCR & IHC	214 samples of OSCC tissues & OC3, OC3IV, CGHNC9 (C9), C9IV3 Oral squamous cancer cell lines	This pathway plays a role in modulating OSCC cancer stemness and drug resistance to chemotherapy.	[253]
PI3-kinase pathway	Tissue Microarray Construction and Immunohistochemistry	123 oral tongue SCC samples	PI3-kinase activation (as determined by PTEN loss) in immune cells infiltrating oral SCC has a novel negative prognostic role.	[254]
Wnt/Ca (2+) /PKC pathway	Western blot	Two different OSCC cell lines	WNT5A activates this non-canonical pathway, resulting in increased migration and invasion of OSCC cells.	[255]
AKT/mTOR Signaling Pathway	flow cytometry & Western Blotting Assay & IHC	Ca9-22 cells & YD-10B cells	Rhein induced the ROS of OC cells to inhibit the AKT/mTOR signaling pathway.	[256]
ANLN/PI3K/mTOR	Western blotting & qPCR	cancer cell lines CAL27 and HN30	ANLN affects the activation of PI3K/mTOR signaling pathway and contributes to OC progression.	[257]
miR-216a-3p and the Wnt-β-catenin signaling pathway	Western blot analysis & Flow cytometric analysis & RT-qPCR & IHC	30 patients with OC and 30 healthy controls & two OC cell lines, HSC-6 and CAL-27	Inhibition of miR-216a-3p inhibits the growth of OC cells, using the Wnt-β-catenin signaling pathway.	[258]
Src/Raf/ERK signaling pathway	flow cytometry & immunoblotting	YD10B and Ca9-22 cells	G-Rh2 inhibits the Src/Raf/ERK signaling pathway in OC by inducing apoptosis and arresting the G0/G1 phase of the cell cycle, thus exerting anti-cancer activity.	[259]
MAPK & Ras/Raf/MEK signaling	Western Blot Assay & DAPI Staining &	OSCC cell lines SAS, SCC9, OECM-1 and HSC3M3	SFB has been shown to decrease MAPK and Ras/Raf/MEK signaling to achieve its pro-apoptotic action. SFB decreases OC cell survival by halting cell cycle at the G2/M phase and causing caspase-mediated apoptosis.	[260]
miR-769-5p / JAK1/STAT3 pathway	western blot assay & flow cytometry assays	OC tissues and cells	miR-769-5p inhibited the growth of OSCC cells by targeting the JAK1/STAT3 pathway, suggesting a potential treatment approach for OSCC.	[261]
LncRNA HNF1A-AS1/ Notch signaling pathway	qrt-pcr & Flowcytometry & Western blotting analysis	62 pairs of human OSCC tissues and their adjacent normal tissues & Five OSCC cell lines (CAL-27, HN5, SCC-15, SCC-9 and Tca8113) and the Keratinocytes of the oral cavity (NHOK)	Transcriptional factor STAT3-induced upregulation of HNF1A-SA1 stimulates the Notch signaling pathway, which in turn advances OSCC.	[262]
circRNA_002178/ Akt/mTOR signaling pathway	qPCR & Western blot	50 pairs of OSCC tissues and adjacent & Human OSCC cells (Fadu, SCC-25, CAL-27, Tca8113) and a normal human oral epithelial cell (Hs 680.Tg)	Due to its overexpression in OSCC tissues and cell lines, CircRNA_002178 may facilitate the malignant development of OSCC by triggering the Akt/mTOR signaling pathway.	[263]

## 5 Spatial metabolomics and transcriptomics in OC research

Single-cell RNA-sequencing (scRNA-seq) is a powerful tool for studying gene activity in different cell types within a tumor. It can reveal how these cells interact with each other. However, there is a catch: scRNA-seq requires breaking the tissue into individual cells, which destroys information about how the cells were initially arranged. This approach makes it difficult to understand the spatial relationships between cells in the tumor microenvironment [[264], [265], [266]]. The heterogeneity of head and neck cancer causes challenges for molecular-level investigation. Spatial metabolomics, a new technique, can analyze the distribution of molecules within a tumor sample and help researchers to understand the biology of the tumor microenvironment. Biomolecules such as proteins, lipids, and metabolites, which show differential expression between tumor and normal tissues, can potentially become diagnostic, prognostic, and therapy [[267], [268], [269], [270], [271]]. Traditional cell studies disrupt tissue structure, while ST and SM preserve spatial data. ST analyzes gene activity directly from tissue sections, preserving this spatial data.

Similarly, SM uses a new type of mass spectrometry to identify and map small molecules within tissues at high resolution. These techniques together provide a more complete picture of how cells and molecules are arranged and function within a tumor [[272], [273], [274], [275], [276], [277]]. Utilizing scRNA-seq data to aid in annotating ST data can significantly augment the sensitivity of the analysis [276,277]. Surgeons struggle to remove all cancer cells during surgery, which can lead to the cancer coming back. Spatial metabolomics is a new technique that can analyze the chemical makeup of tissue to tell the difference between healthy and cancerous tissue. Studies have shown success using this technique in brain, stomach, pancreas, and breast cancers. These advancements underscore spatial metabolomics' potential to improve diagnosis and treatment precision for head and neck tumors, ultimately benefiting patient outcomes [[278], [279], [280], [281], [282], [283], [284]]. ST and SM provide a comprehensive understanding of tumor biology, aiding in diagnosis, treatment planning, and patient outcomes for head and neck cancers. Combining these technologies with single-cell RNA sequencing enhances analysis sensitivity.

## 6 OC treatment methods and therapeutic potential of non-coding RNAs in OC

Various treatments are available for OSCC, depending on the patient's general health and the size, location, and tumor stage.

### 6.1 Surgical Resection

Surgical surgery is the primary therapeutic option for OC patients, particularly those in advanced stages of the disease [285]. Surgery is the best treatment for tumors that can be removed; however, to perform a surgical resection, there must be enough margins available for the original tumor. A poor prognosis results from an increased risk of local recurrence caused by an inability to obtain a clear surgical margin [286,287].

### 6.2 Chemotherapy

Chemotherapy is a systemic treatment that can cause severe nausea and gastrointestinal distress as well as pancytopenia [288]. Cancers that cannot be surgically removed due to their size or dispersion can be treated with chemotherapy. Chemotherapy helps to treat any cancer-related symptoms and delays the spread of the disease for as long as feasible. Cisplatin, carboplatin, 5-fluorouracil, paclitaxel, docetaxel, and hydroxyurea are the most often used chemical medications for OC. These medications can be used on their own or in combination with other medications [289,290]. Chemotherapy efficacy is also hindered by the high osmotic pressure of tumor tissue, which makes it difficult for chemotherapy medications to enter the tumor site. On the other side, cancer is easily resistant to chemotherapy medications, making treatment more difficult [291].

#### 6.2.1 Non-coding RNAs in chemotherapy and drug resistance

MiRNAs, circRNAs, and lncRNAs make up the majority of ncRNAs involved in chemoresistance [292,293]. Recent research has demonstrated the role miRNAs play in OC drug resistance. Compared to normal human oral keratinocytes, Chang et al. discovered that OSCC cancer cell lines and primary cultures expressed more Twist and Snail and less let-7d. Furthermore, overexpression of let-7d significantly reduced OSCC cells' resistance to CDDP (cisplatin) and 5-fluorouracil (5-FU) [294].

**Figure 5:**
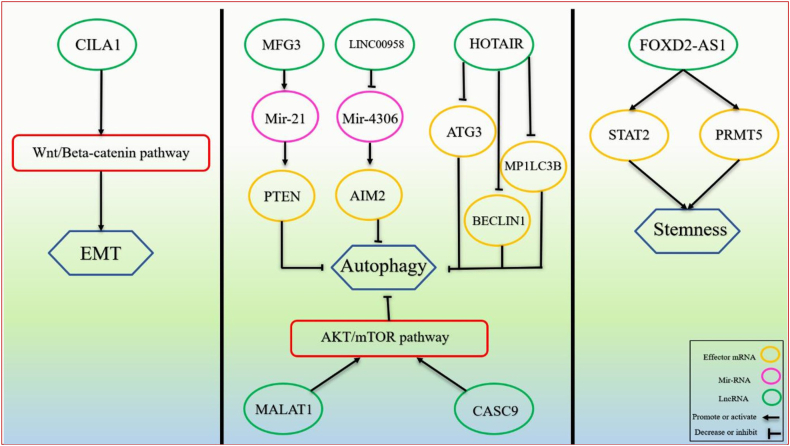
A summary of lncRNA molecular pathways involved in OC treatment resistance. The three most essential cellular mechanisms involved in drug resistance are described: autophagy, EMT, and stemness.

LncRNAs control every biological activity, including apoptosis, drug efflux systems, drug metabolism, DNA repair, EMT, and autophagy (figure. 5). They are also recognized as essential regulators of tumors and carcinogenesis and possible mediators of drug resistance. In addition, lncRNAs play an essential role in HNC cell resistance to chemotherapy [295,296]. Some of these ncRNAs are summarized in the table 7.

**Table 7:** Some of the researches done in the field of the role of ncRNAs in treatment resistance

Non-coding RNA	Sample	Expression	Function	reference
miRNA let-7d	OECM1, primary cell	-	decrease the expression of Twist, Snail / Corresponding drugs: CDDP,5-FU	[294]
miR-21	HSC, SCC-9 cell lines	+	decrease the expression of PTEN and PDCD4 / Corresponding drugs: CDDP	[297]
miR-654-5p	Tca8113, CAL27 cell lines	+	through Ras/MAPK signaling pathway/ Corresponding drugs: CDDP,5-FU	[298]
miR-365-3p	OC3, CGHNC9 cell lines	+	enhancing β5-integrin/c-met signaling pathway / Corresponding drugs: 5-FU	[299]
miR‐221	SCC‐4 and SCC‐9 OSCC cells	+	Anti‐apoptosis; Targets TIMP3 axis / Corresponding drugs: doxorubicin	[300]
lncRNA /ANRIL	OSCC‐3, SCC‐4, HSC‐3 and CAL‐27 cells; OSCC tumor tissues	+	Promotes proliferation; Targets MK/ANRIL/MRP1 and ABCC2/ caspase‐3/BCL‐2 axis / Corresponding drugs: CDDP	[301]
lncRNA /HOTAIR	KB, CAL-27 cell lines	+	decreases the CDDP sensitivity / Corresponding drugs: CDDP	[302]
lncRNA/ UCA1	Tca8113, TSCCA, CAL‐27 and SCC‐9 cells; OSCC tumor tissues	+	Promotes proliferation; Targets UCA1/miR‐184/SF1 axis / Corresponding drugs: CDDP	[303]
Circ ITCH	SCC‐6, SCC‐9, SCC‐25, HN‐4 and HN‐6 cells; OSCC tumor tissues	-	Promotes proliferation; Targets miR‐421/PDCD4 Axis / Corresponding drugs: Bortezomib	[304]

### 6.3 Radiation Therapy

Brachytherapy and external beam radiation are two methods that can be used for OSCC treatment. External beam radiation therapy uses specialized equipment, such as betatrons and linear accelerators, to produce high-energy radiation, such as X-rays, that, when directed towards the cancer site from the outside, can destroy cell chromosomes and stop cell proliferation, killing cancer cells with a high rate of growth and division. Traditionally, brachytherapy implants use iridium-192 needles or iodine-125 seeds to deliver high-dose radiation to specific regions while sparing the surrounding healthy tissues. To kill mitotic cells by damaging DNA, radiation therapy is frequently employed in conjunction with surgery or chemotherapy [[305], [306], [307]].

### 6.4 Targeted Therapy

The U.S. Food and Drug Administration (FDA) approved the EGFR antagonist cetuximab monoclonal antibody in 2006, making it the only first-line targeted therapy for OSCC. Cetuximab's ability to prevent EGF and EGFR from binding together can disrupt the downstream reaction brought on by EGFR activation. Moreover, cetuximab improves radiation therapy-assisted local tumor management. Nevertheless, patients are likely to experience drug resistance in the later stages of cetuximab treatment, which reduces the effectiveness of the clinical intervention [308,309].

### 6.5 Immunotherapy

Immunotherapy is a novel approach to tumor treatment that enhances the targeted immune response to the tumor by using immunological techniques and biotechnology [310]. Immunotherapy is a successful cancer treatment that controls and eradicates tumor cells because of its capacity to suppress immune cells in the tumor microenvironment and activate the body's immune system. Tumors use particular immune checkpoint pathways as their principal immunological resistance strategy, primarily against T cells that target tumor antigens. The PD-1/PD-L1 pathway, which is targetable, is one significant method that tumors avoid the immune system [311]. A checkpoint for immunity on the surface of T cells is called programmed cell death-1 (PD-1). Induced PD-1 expression occurs during T cell activation. PD-1 can attach to its ligand, PD-L1, on tumor cells, protecting them against autoimmunity-induced apoptosis and allowing PD-1 to block the PD-1 pathway and suppress T cell immune response [312]. Anti-PD1/PD-L1 drugs increase the anti-tumor immune response by blocking the immunosuppressive signaling of tumors. The FDA has approved nivolumab and pembrolizumab as the first two anti-PD-1 monoclonal antibodies [313].

Tumor vaccine therapy, adoptive cell immunotherapy, antibody-based therapy, cytokine therapy, and gene therapy are just a few of the immunotherapy applications with exceptional utility in tumor treatment [314].

#### 6.5.1 Non-coding RNA in immunotherapy

Several immune-related lncRNAs are hypothesized to have regulatory functions in immune processes at the epigenetic level. LncRNAs control T and B lymphocyte activation and differentiation during the adaptive immune response. LncRNAs control innate immune cell function and inflammatory cytokine production [315]. By regulating immune cell homeostasis, function, and anti-inflammatory substances, lncRNAs can modulate immunological responses. Consequently, immune-related lncRNAs are the term given to these molecules [316,317]. A significant contributing factor to immunotherapy resistance may be the immunosuppressive environment lncRNAs might create through their ability to modify different T cell-mediated immune responses [318].

Tumor cells prefer to use glycolysis, which produces many lactates, to generate energy due to mitochondrial failure and the aerobic environment. The acidification of the TME caused by lactate generated by aerobic glycolysis can impair immune cell activity and prevent the release and release of several anticancer cytokines. Cells use membrane proteins called glucose transporters, or GLUTs, to move glucose around. Aerobic glycolysis and glucose transport into cells are enhanced by the aberrant expression of GLUT on the surface of tumor cells. It has been discovered that ncRNAs regulate GLUT in human malignancies [319,320]. In oral squamous cell carcinoma, for instance, miR-340 downregulates GLUT1 expression; [321] lncRNA p23154 promotes GLUT1 expression; [132], and miRNA-375 downregulates LDHA and inhibits aerobic glycolysis [322].

### 6.6 Phytochemicals Therapy

Natural biologically active phytochemicals have drawn attention recently due to their possible health benefits. These phytochemicals are excellent chemopreventive agents because they are either very innocuous to healthy tissues or not at all. However, the typical low water solubility, low bioavailability, and inadequate targeting of phytochemicals restrict their potential therapeutic applications [323].

Through a variety of methods, including the inhibition of cancer spread, the targeting of cancer stem cells (CSCs), the enhancement of human immunity, antioxidative activity, the regulation of epigenetics and epigenomics, and anti-inflammatory responses, phytochemicals have been demonstrated to possess anticancer capabilities. Additional effects include encouraging apoptosis in cancer cells, preventing the advancement of the cancer cell cycle, and blocking cell signal transmission, eventually preventing cancer cells' growth and angiogenesis. Cell cycle arrest, suppression of proliferation, and induction of apoptosis—all brought about by the down-regulation of cell cycle regulators such as cyclins—are important mechanisms behind these effects [[324], [325], [326], [327], [328], [329], [330], [331], [332]]. Table 8 lists some clinical studies for OC.

**Table 8:** The clinical trials in OC

no	NCT Number	Study Title	Status	Study Type	Study Start
1	NCT05003427	68Ga-FAPI-04 PET/CT for the Detection of Oral Carcinoma	Recruiting	Interventional	2021-04-01
2	NCT00172965	Diagnosis of Oral Precancers and Cancers Using Optic Coherence Tomography	Unknown status	Observational	2004-08
3	NCT04398121	NBI in Oral Cavity Cancer	Completed	Observational	2020-08-13
4	NCT04330781	Radiotherapy of Head and Neck Cancer Using an Intraoral Stent	Recruiting	Interventional	2020-01-01
5	NCT05902455	Differential Mobility Spectrometry (DMS) Based Oral Tumor Analysis	Recruiting	Observational	2021-05-25
6	NCT03853655	Adjuvant Radiotherapy in EarlyStage OCs	Active, not recruiting	Interventional	2018-08-02
7	NCT00173316	Expression of VEGF-C and VEGF-CR in OCs and Premalignant Lesions	Unknown status	Observational	2004-08
8	NCT00154973	Expression of Hypoxia-Inducible Factor-α in Oral Precancers and Cancers	Unknown status	Observational	2004-08
9	NCT03545100	Rehabilitation Outcomes of Shoulder Function in OC Survivors Cancer Survivors	Completed	Interventional	2018-06-01
10	NCT04925700	The Oral Microbiome in OSCC	Completed	Interventional	2022-04-20
11	NCT04913545	The Senstivity and Specificity of Using Salivary miRNAs in Detection of Malignant Transformation of Oral Lesions.	Completed	Observational	2019-06-05
12	NCT04305366	MicroRNA Markers in Head and Neck Cancers	Active, not recruiting	Observational	2012-11-16
13	NCT05708209	The Long Non-Coding MALAT1 as a Potential Salivary Diagnostic Biomarker in Oral Squamous Cell Carcinoma Through Targeting mi RNA 124	Completed	Observational	2022-11-01
14	NCT04946968	Phase-2 Dacomitinib Study on Patients With EGFR-Driven Advanced Solid Tumours with Low EGFR-AS1 IncRNA Expr or Other Novel Emerging Biomarkers	Recruiting	Interventional	2021-08-24
15	NCT05708209	The Long Non-Coding MALAT1 as a Potential Salivary Diagnostic Biomarker in Oral Squamous Cell Carcinoma Through Targeting mi RNA 124	Completed	Observational	2022-11-01

## 7 Conclusions and Future Perspectives

Despite treatment options like surgery, radiation, and medications, the outlook for advanced OC remains grim. This is reflected in the high number of deaths attributed to this disease every year (around 180,000) and its negative impact on patients' daily lives [333,334]. Advancements in transcriptome profiling have deepened our understanding of cancer and transformed it into a data-intensive scientific domain. Technological breakthroughs enable higher-resolution RNA profiling across spatial, temporal, and molecular aspects while decreasing sequencing costs make high-throughput analyses more accessible [74]. CRISPR-Cas9-mediated editing of lncRNAs has been widely used in various diseases, including cancer. Although insufficient research confirms its effectiveness for oral squamous cell carcinoma (OSCC), theoretical connections suggest promising potential [335,336]. Researchers have identified specific molecules within exosomes that could serve as prognostic tools and potential therapeutic targets.

For instance, salivary exosomal miRNA-1307-5p is associated with poor patient outcomes in OC. Additionally, studies revealed that lncRNAs like TIRY, MAGI2-AS3, and CCDC144NL-AS1 carried by exosomes can influence OC progression and metastasis by impacting factors like miR-14 expression and the PI3K-AKT-mTOR pathway, highlighting their potential role in disease development [156,337,338]. MALAT-1, a molecule found within tiny packages called exosomes released by tumors, can promote cancer growth and spread [339]. Non-coding RNAs (ncRNAs) such as miRNAs, lncRNAs, and circRNAs show promise as salivary biomarkers, and Let-7d are potent miRNAs for detecting metastatic potential. In contrast, lncRNAs like MALAT1 and HOTAIR influence disease progression through crucial signaling pathways. CircRNAs like hsa_circ_0001874 and hsa_circ_0001971, which functionally complement miRNAs, are significant in disease progression [340,341]. NcRNAs are emerging as powerful tools in precision medicine for oral inflammatory diseases. Their influence on DNA translation, mRNA and protein regulation, and cellular signaling pathways makes them ideal candidates for several clinical applications. ncRNAs can be used for disease diagnosis and prognosis, allowing for more targeted treatment approaches [342]. Despite advancements in treatment options such as surgery, radiation, and medication, the prognosis for advanced OC remains a significant health challenge with a high mortality rate. Integrating genomic and transcriptomic data provides a comprehensive view of tumor biology, highlighting potential avenues for precision medicine. Future research on ncRNAs and their regulatory functions could pave the way for innovative diagnostic tools and tailored therapeutic strategies, ultimately improving outcomes for patients with OC. These advancements highlight a shift towards a more precise and data-driven approach to diagnosing and treating OC.

## CRediT authorship contribution statement

**Mehrdad Hashemi:** Writing – original draft, Visualization, Conceptualization. **Saloomeh Khoushab:** Writing – original draft, Conceptualization. **Mina Hobabi Aghmiuni:** Writing – original draft, Conceptualization. **Saeid Nemati Anaraki:** Visualization, Resources, Investigation. **Mina Alimohammadi:** Writing – review & editing. **Afshin Taheriazam:** Supervision. **Najma Farahani:** Writing – review & editing, Supervision. **Maliheh Entezari:** Writing – review & editing, Supervision.

## Data and code availability statement

No data was used for the research described in the article.

## Declaration of competing interest

The authors declare that they have no known competing financial interests or personal relationships that could have appeared to influence the work reported in this paper.
